# ER-phagy mediates the anti-tumoral synergism between HDAC inhibition and chemotherapy

**DOI:** 10.1186/s12964-025-02198-9

**Published:** 2025-04-26

**Authors:** Felix J. Gössl, Pierfrancesco Polo, Frederik Helmprobst, André Menzenbach, Alexander Visekruna, Thomas M. Gress, Till Adhikary, Matthias Lauth

**Affiliations:** 1 Clinic of Gastroenterology, Endocrinology and Metabolism, Center for Tumor- and Immune Biology, Philipps University Marburg, Hans-Meerwein-Str. 3, Marburg, 35043 Germany; 2 Core Facility for Mouse Pathology and Electron Microscopy, Philipps University Marburg, Marburg, 35043 Germany; 3 Institute for Medical Microbiology and Hygiene, Philipps-University Marburg, Marburg, 35043 Germany; 4 Center for Tumor- and Immune Biology, Institute for Biomedical Informatics and Biostatistics, Philipps University Marburg, Hans-Meerwein-Str. 3, Marburg, 35043 Germany

**Keywords:** ER-phagy, FAM134B, RETREG1, HDAC inhibitors, ER-stress, Chemotherapy, Gemcitabine, Pancreatic cancer

## Abstract

**Background:**

Histone deacetylase inhibitors (HDACi) are clinically approved drugs for the treatment of hematological malignancies synergizing with chemotherapy. However, despite the long history of HDACi, the mechanistic underpinnings of this synergism have remained unclear.

**Methods:**

Using transmission electron microscopy, we identified autophagy and ER-stress in HDACi-treated cells. We quantified ER-phagy and ER-stress with reporter systems by using 3D-deconvolution microscopy and flow cytometry. We complemented these data with qPCR and Western blot results. Apoptosis rates were assessed using a caspase assay and flow cytometry, and large public datasets were utilized.

**Results:**

HDAC blockade results in specific upregulation of the selective autophagy receptor FAM134B (RETREG1) and the induction of ER-phagy. Combined with the chemotherapeutic drug Gemcitabine, this results in subsequent elevated ER-stress levels and apoptosis. Inhibiting the distinct ER-stress branches fully rescues this process. Broadening the scope of these findings, certain non-HDAC-inhibitory and clinically approved compounds like Loperamide and Nelfinavir are able to induce FAM134B and could hence constitute novel Gemcitabine-synergizing molecules. Additionally, pancreatic cancer patients with high *FAM134B* expression have significantly longer survival rates under chemotherapy.

**Conclusion:**

In summary, we provide mechanistic evidence for ER-phagy playing a hitherto unknown central role in the clinical synergy between HDACi and chemotherapy.

**Supplementary Information:**

The online version contains supplementary material available at 10.1186/s12964-025-02198-9.

## Introduction

Pharmacological blockade of histone deacetylation by selective inhibitors (HDACi) has been approved for the treatment of lymphomas and myeloma [1–3]. Moreover, it is well-established that HDACi sensitize cancer cells to chemotherapy, a phenomenon that has also been demonstrated in numerous pre-clinical models of solid cancer [4–6]. This also holds true for pancreatic cancer (pancreatic ductal adenocarcinoma; PDAC), where HDACi synergize with Gemcitabine, a frequently used drug in the treatment of this highly aggressive malignancy [7–9]. Given that chemoresistance poses a significant challenge in the therapy of many cancers, including PDAC [10], the synergism between HDACi and chemotherapy/Gemcitabine is of high clinical interest. However, although HDAC inhibitors have been known for many years, the cellular mechanisms underlying this pharmacological cross-talk have remained elusive [11].

Using a recently discovered novel HDAC-inhibiting small molecule [12, 13] as well as approved HDACi, together with detailed electron microscopy studies, we observed evidence for autophagic digestion of endoplasmic reticulum (ER-phagy; a.k.a. reticulophagy) and ER-stress in treated PDAC cells. Further investigations revealed that HDAC-inhibitory compounds preferentially upregulate the ER-phagy receptor *Family with sequence similarity 134, member B* (*FAM134B;* a.k.a. *RETREG1*), compared to other selective autophagy receptors targeting the ER, resulting in the induction of ER-phagy. This leads to subsequent ER-stress, which is further potentiated by Gemcitabine and which shows an activation of all three major ER signaling pathways (i.e. the inositol-requiring enzyme 1 (*IRE1*, a.k.a. *ERN1*)-, the protein kinase R (PKR)-like ER Kinase (*PERK*, a.k.a. *EIF2 AK3*)-, and the activating transcription factor 6 (*ATF6*)-branches). The synergistic elevation of ER-stress by HDACi and Gem caused a significant upregulation of *C/EBP homologous protein* (*CHOP*; a.k.a. *DDIT3*), eventually causing apoptosis. Interestingly, whereas ER-stress was highly dependent on extracellular calcium, the induction of *FAM134B* was not. Moreover, blocking ER-stress completely abrogated the synergistic effects of HDACi and Gem.

In addition, we further observed that the entirely unrelated and non HDAC-inhibitory anti-diarrheal drug Loperamide, as well as the anti-viral compound Nelfinavir, could also induce the ER-phagy receptor *FAM134B*. This underscores the importance of the ‘FAM134B→ ER-phagy→ ER-stress→ apoptosis’ axis in the treatment of cancer, also with clinically approved drugs. Focusing on prognostic and predictive relevance, we could show that PDAC patients with high expression of *FAM134B* displayed a significantly longer overall survival, suggesting that ER-phagy could represent a novel biomarker for chemotherapy response and survival. Taken together, we could shed light on the interaction between HDACi and chemotherapy and could place ER-phagy in the center of this synergism.

## Material and methods

### Cell lines

The following cell lines were used: Panc1 (ATCC, CRL- 1469) and MIA PaCa- 2 (ATCC, CRL- 1420). All cell lines were cultured in Dulbecco’s Modified Eagle Medium (DMEM (high Glucose plus Glutamine and Pyruvate), ThermoFisher, 41,966,029), supplemented with 10% fetal bovine serum (FBS constance, Anprotec, AC-SM- 0190) and 1% Penicillin/Streptomycin (Capricorn Scientific, PS-B) at 37 °C with 5% CO_2_. If not otherwise stated, serum concentrations were reduced to 0.5% during experiments for all cell types. All cells were regularly checked for mycoplasma contamination.

### Reagents

The following chemicals were used: Ceapin-A7 (Sigma Aldrich/Merck, SML2330), Chidamide (Cayman Chemical Company, 13,686), Gemcitabine (Sigma Aldrich/Merck, G6423), Hygromycin B (Angene, AG00GAHS), Isoxazole 9 (ISX) (Biomol, 16,165), ISRIB (Sigma Aldrich/Merck, SML0843), Loperamide (LKT labs, L5660), Nelfinavir Mesylate (Cayman Chemical Company, 15,144), MS- 275 (AdipoGen Life Sciences, AG-CR1 - 0032), Puromycin dihydrochloride (PAA, P15 - 019), Staurosporine (Cayman Chemical Company, 81,590), 4μ8c (Cayman Chemical Company, 22,110), Thapsigargin (Cayman Chemical Company, AG-CN2 - 0003).

### Antibodies and plasmids

The following antibodies were used: FAM134B (Cell Signalling Technology, 83,414), β-Actin (Sigma Aldrich/Merck, A5441), anti-mouse IgG HRP conjugated (Cell Signalling Technology, 7076 s), anti-rabbit IgG HRP conjugated (Cell Signalling Technology, 7074 s). The following plasmids were used: IRE1 reporter/pLHCX-XBP1 mNeonGreen NLS (Addgene 115971; David Andrews), PERK reporter/pLHCX-ATF4 mScarlet NLS (Addgene, 115,970; David Andrews), ER-Keima reporter/GST-Keima-cb5 (Addgene, 137,755; Carol Mercer), mCherry-LC3B (Addgene 40827, David Rubinsztein), ATF6 reporter/p5xATF6-GL3 (Addgene, 11,976, Ron Prywes). The negative control plasmid p0xATF6-Luc was generated by us by removing the five ATF6 binding sites. The Renilla plasmid (R-Luc) for normalization was pRL-TK from Promega.

### Cell titer assays

Cell titer assays were performed by seeding 10.000 cells in each well of a white 96-well plate with a clear bottom and treating them for 4 days in 0.5% FBS-containing growth medium. Subsequently, cell titers were determined using the Cell Titer-Glo® assay kit (Promega, G9241) and a Microplate Luminometer (Berthold Orion II).

### RNA/cDNA analysis

According to the manufacturer's protocol, total RNA was extracted using the NucleoSpin RNA II kit (Macherey–Nagel, 740,955.50). 1 µg of total RNA was used for cDNA Synthesis using AB Script II cDNA Synthesis Kit (ABclonal, RK20400). The Absolute QPCR SYBR Green Mix (ABclonal, RK20400) was used for quantitative PCR reactions. qPCR reactions were performed using either the Mx3000P (Agilent Technologies) or Quantabio Q 4 channel (Quntabio). Relative expression was calculated according to the 2^−ΔΔCt^- method.

### Western blotting

Lysates were separated by SDS-PAGE (Bio-Rad) and subsequently blotted on Immobilon-PVDF membranes (Merck, IPVH00010). This was followed by incubation with the respective primary antibody and a corresponding HRP-coupled secondary antibody, as described in [14]. According to the manufacturer's protocol, the HRP signal was detected using Pierce ECL Western Blotting Substrate (ThermoFisher, 32,106).

### Microscopy

Cells were seeded on etched coverslips and treated according to the respective experimental protocol. Subsequently, cells were fixed in 4% formaldehyde/PBS (10 min at RT), washed, permeabilized with 0.5% Triton-X100 (Sigma Aldrich/Merck, X100) and blocked with 10% FBS/PBS for 1 h at RT. Then, cover slips were incubated with the primary antibody in PBS containing 10% goat serum (Sigma Aldrich/Merck, G6767) and 0.1% Saponin (Sigma Aldrich/Merck, 8047–15 - 2) overnight at 4 °C. After washing with PBS (ThermoFisher, 14,190) at RT, cover slips were incubated with fluorophore-coupled secondary antibodies diluted in antibody-solution (PBS, 10% goat serum, 0.1% Saponin) at RT in the dark for 2 h. After washing with PBS and rinsing with water, coverslips were mounted with a mounting medium containing DAPI/Vectashield (BIOZOL, H- 1200). Immunofluorescence images were recorded using a Widefield Microscope (Leica DM 5500 B; Leica Microsystems, Wetzlar, Germany), and z-stacks were deconvoluted using Leica LAS AF 4.0.

### Transfection

600.000 cells were seeded in a 6-well plate in the morning; in the evening, they were transfected using TransIT®− 2020 (Mirus Bio, MIR 5404) according to the manufacturer’s protocol. Stable transfections: Cells transfected with a reporter were selected for two weeks using Hygromycin B (100 μg/ml; 190 μM) for the IRE1- and PERK-reporter and Puromycin dihydrochloride (1.0 μg/ml; 1.8 μM) for the mKeima-reporter. The *in-house* FACS Core Facility sorted these cells using an Aurora CS (Cytek). Sorted cells were cultured in complete growth medium containing the respective antibiotic.

### Flow cytometry

500.000 cells were treated in 6-well plates. These cells were subsequently trypsinized, resuspended in DMEM, washed twice with MACS Buffer, and clumps were sieved. Samples were analyzed using the FACS Canto II (BD), CytoFLEX LX Series (Beckman Coulter) or FACSCelesta (BD). Untreated and untransfected cells were included as negative controls. For analysis of the resulting FCS files, we used FlowJo v10.10 software. For the analysis of the ER-Keima reporter, the ratio of autolysosomes derived from the ER (Ex. λ = 633 nm, gMFI(APC-A) was divided by neutral ERs (Ex. λ = 488 nm, gMFI (FITC-A)) and the value of DMSO was subtracted.

### Apoptosis assays

Apoptosis assays were performed by seeding 10.000 cells in each well of a white 96-well plate with clear bottom. These cells were treated for four days in 5% FBS-containing growth medium. Subsequently, apoptosis rates were determined using the Caspase Glow® 3/7 kit (Promega, G8090) in a Microplate Luminometer.

### Annexin V apoptosis detection with PI

Two hundred thousand Panc1 cells were seeded in a 12-well plate in the evening and treated the following morning. After 60 h, the cells were washed with Annexin V 1 × binding buffer (10 mM HEPES/NaOH, 140 mM NaCl, 2.5 mM CaCl_2_, 0.1% BSA (Roth, 8076.3), pH 7.4), trypsinized, and then washed again. Cells were stained with 3 μl Annexin V-FITC (Miltenyi Biotec, 130–092 - 052) in 100 μl Annexin V 1 × binding Buffer for 15 min at room temperature (RT). After another wash, the cells were stained with 1 μl Propidium Iodide (AAT Bioquest, 17,585; 1 mg/ml) in 100 μl of Annexin V 1 × binding buffer for 2 min at RT, washed again, sieved to remove clumps, and analyzed using flow cytometry.

### Luciferase assays

One hundred fifty thousand cells were transfected overnight using TransIT- 2020 (Mirus, MIR 5404) with either p5xATF6-Luc or p0-ATF6-Luc missing ATF6 binding sites. Additionally, all cells were transfected with R-Luc plasmid for normalization. Transfected cells were re-seeded in the morning and treated in the evening for 2 d. Cells were washed and lysed with passive lysis Buffer 1x (Promega, E1910). Each sample was measured in technical triplicates using a 96-well microplate, with Renilla-Juice Luciferase Assay and Beetle-Juice Luciferase Assay Firefly (PJK GmbH, 102,531) in a Microplate Luminometer. A double normalization was performed on both, the Renilla signal and on DMSO values.

### TEM

Five hundred thousand cells were treated for 48 h in a 6-well plate. Cells were fixed with glutaraldehyde, OsO_4,_ and contrasted with uranyl acetate, according to the literature [15]. TEM pictures were analyzed using 3 dmod blindly to avoid confirmation bias. The original publication [16] describes an early version of the 3 dmod/IMOD software.

### Statistics

If not stated otherwise, statistical comparisons were made of *n* ≥ 3 experiments using an unpaired two-tailed Student’s t-test (GraphPad Prism). Significances were indicated as ns (not significant; *p* ≥ 0.05), *p < 0.05, **p < 0.01, ***p < 0.001. Kaplan–Meier curves were generated using the R2: Genomics Analysis and Visualization Platform (http://r2.amc.nl).

## Results

### HDAC inhibition promotes ER-phagy

**Fig. 1:**
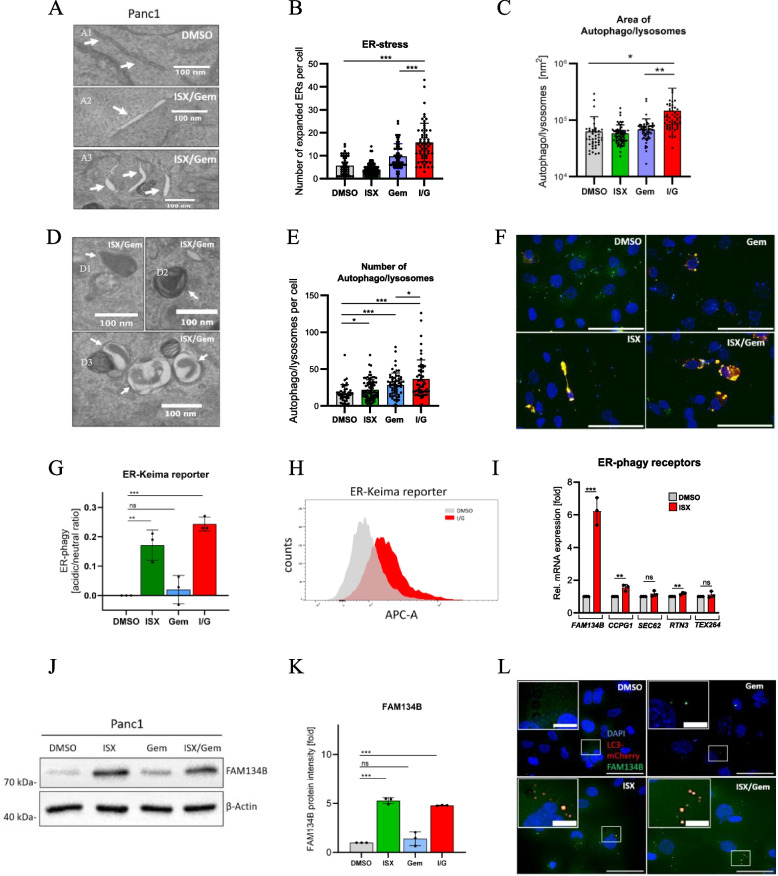
ISX induces ER-phagy (**A**) Magnified TEM pictures showing ER. Panc1 cells were treated for 48h with DMSO, 20 µM ISX, and 1.0 µM Gem. Arrows indicate normal (A1) or dilated (A2, A3) ER. (**B**) Quantification of ER stress by measuring the number of expanded ER per Panc1 cell in each treatment group as shown in (A). I/G=ISX/Gem. Each dot represents one cell (mean ± SD). (**C**) The area of autophago- and autolysosomes per cell. Each dot represents one cell (mean ± SD). (**D**) Magnified TEM pictures showing autophagosomes (D1) and autolysosomes (D2, D3) in double-treated cells (ISX 20 µM, Gem 1.0 µM for 48h). (**E**) Number of autophago- and autolysosomes per Panc1 cell. Each dot represents one cell (mean ± SD). Treatment as in (**A**). (**F**) Immunofluorescence pictures of Panc1 cells transiently transfected with ER-Keima reporter and treated as described in (**A**). The pictures shown are overlay images depicting non-phagocytosed ER (green) and ER-derived autolysosomes (red). Scale bar: 60µm. Shown is one representative experiment of n=2. (**G**, **H**) Flow cytometric quantification of the ER-phagy in Panc1 cells stably expressing ER-Keima reporter, treated as described in (**A**) (mean of n=3 ± SD) and histogram of flow cytometry. (**I**) Relative mRNA expression of ER-phagy receptors (*FAM134B*, *CCPG1*, *SEC62*, *RTN3*, *TEX264*) in Panc1 cells treated for 48h with DMSO or 20 µM ISX (mean of n=3 ± SD). (**J**) Western blot analysis of FAM134B levels in Panc1 cells treated for 48h as described in (A). β-Actin was used as a loading control. One representative of n=3 is shown. (**K**) Quantification of FAM134B protein levels as depicted in (**J**) (mean of n=3 ± SD). (**L**) Colocalization of mCherry-LC3B (red) and endogenous FAM134B (green) of Panc1 cells treated for 48h. Treatment as in (**A**). One representative of n=4 is shown. Scale bar: 50µm in the larger panel and 10µm in the magnified insets

In order to investigate the impact of the HDACi/chemotherapy synergism on cancer cell biology in more depth, we treated PDAC cells with Gemcitabine (Gem) and the small molecule Isoxazole 9 (ISX) [17], which we recently identified as a novel non-canonical inhibitor of cellular HDACs [13]. As expected, ISX behaved similarly to clinically approved class I HDACi such as Chidamide (a.k.a. Tucidinostat) or MS- 275 (a.k.a. Entinostat) in synergizing with Gemcitabine in killing Panc1 cancer cells (Fig. S1 A). These experiments were performed in low serum conditions to mimic the nutrient-poor microenvironment of PDAC. We previously proved that these conditions did not affect basal levels of apoptosis (Fig. S1B,C), which might have impacted cell killing rates by HDACi. Next, using transmission electron microscopy (TEM) to study the synergism of ISX and Gem in more detail, we remarkably observed that the drug combination resulted in significantly increased numbers of dilated endoplasmic reticulum (ER) structures, a typical feature of ER-stress (Fig. 1A,B). In addition, ISX/Gem treatment increased the size and numbers of autophagosomes/autolysosomes, which often contained membranous elements resembling ER (Fig. 1C,D,E). The engulfment of ER into autophagic vesicles is a cardinal sign of ER-phagy, the self-digestion of ER for the purpose of remodeling [18, 19]. We used an ER-phagy reporter (ER-Keima), which changes its excitation maximum to longer wavelengths when acidified within autophagic vesicles when ER-phagy occurs [20]. We obtained microscopic evidence for ER-phagy in ISX-exposed cells, which was further enhanced upon co-treatment with Gem (Fig. 1F). A clinically approved HDACi showed similar results (Fig. S1D). Next, we quantified ER-phagy using ER-Keima reporter and flow cytometry. We detected a significant ER degradation in the ISX and ISX/Gem conditions (Fig. 1G,H), as well as with approved HDACi (Fig. S1E). Notably, there was no significant ER degradation with Gem treatment alone (Fig. 1G).

Selective autophagy, such as ER-phagy, is mediated by specific adapter molecules functioning as receptors recruiting the ER to the autophagic machinery. We, therefore, screened the expression of known ER-phagy receptors upon ISX and Gem treatment. Strikingly, ISX selectively induced the expression of *FAM134B,* with only little effect on additional ER-phagy receptors: *Cell cycle progression 1* (*CCPG1*), *SEC62 homolog, preprotein translocation factor* (*SEC62*), *Reticulon 3* (*RTN3*) and *Testis expressed 264 ER-phagy receptor* (*TEX264*) (Fig. 1I,J,K). Similar findings were obtained with clinically approved HDACi (Fig. S1 F). Importantly, *FAM134B* induction was not limited to Panc1 cells but was also found in the PDAC cell line MIA PaCa- 2 (Fig. S1G). Notably, FAM134B protein co-localized with the general autophagy mediator LC3B in treated cells, indicative of ongoing ER-phagy (Fig. 1L). Equivalent findings were obtained with a clinically approved HDACi (Fig. S1H). In summary, we could provide evidence for HDACi inducing the selective ER-phagy receptor *FAM134B*. Individual treatment with HDACi induces ER-Phagy, while the combination with Gem enhances this effect.

### ISX/Gem causes broad ER-stress

**Fig. 2:**
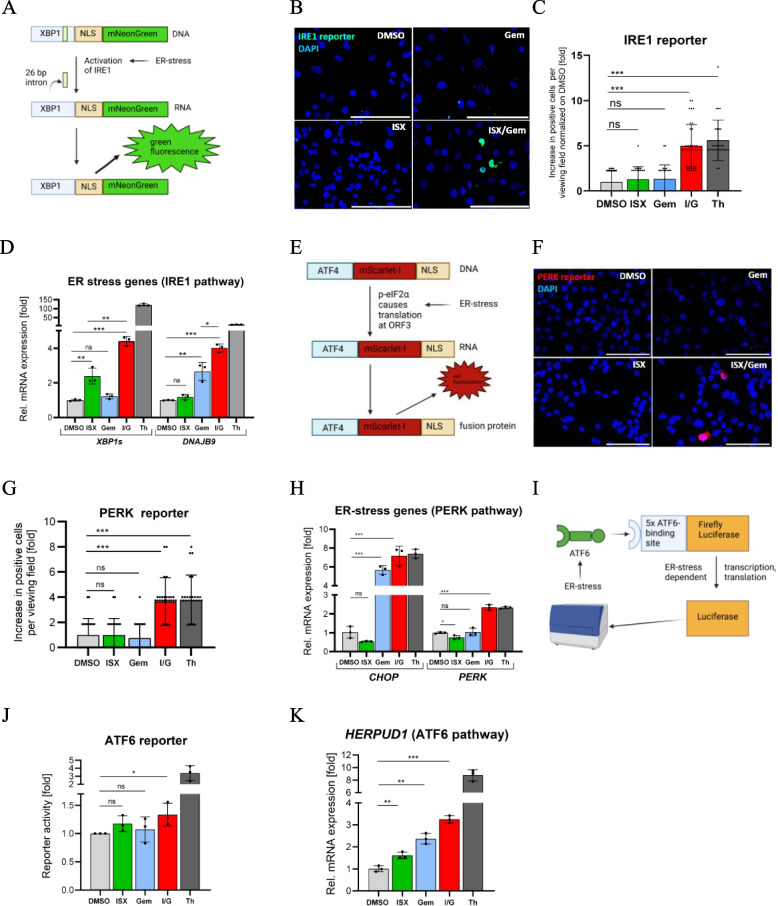
ISX and Gemcitabine synergistically induce ER-stress (**A**) Scheme of the IRE1-selective ER-stress reporter. (**B**) Immunofluorescence images of Panc1 cells transiently transfected with IRE1 reporter (green), treated with DMSO, 20 µM ISX, 1.0 µM Gem and the combination ISX/Gem (I/G) for 48h. Nuclei appear blue (DAPI). Scale bar: 100µm. Shown is one representative of n=3. (**C**) Quantification of Panc1 cells transiently expressing IRE1 reporter. Cells were treated as described in (**B**) or with 1.0 μM Thapsigargin (Th) for 48h, analyzed using the number of positive cells per viewing field normalized to the values of the DMSO samples. Each dot represents one image (from n=2 independent experiments; mean ± SD). (**D**) Rel. mRNA expression of ER-stress genes selective for the IRE1 pathway (*XBP1s*, *DNAJB9*). Panc1 cells were treated as described in (**C**) (mean of n=3 ± SD). (**E**) Scheme of the PERK-selective ER-stress reporter. (**F**) Immunofluorescence pictures of Panc1 cells transiently expressing PERK reporter (red) and treated as in (**B**). Nuclei appear blue (DAPI). Scale bar: 100µm. Shown is one representative of n=3. (**G**) Quantification of PERK pathway activity in Panc1 cells stably expressing a PERK reporter construct. Treatment as in (**C**). Shown are the numbers of positive cells per viewing field normalized to the DMSO condition. Each dot represents one analyzed viewing field (mean ± SD of n=2). (**H**) Rel. mRNA expression of ER-stress genes selective for the PERK pathway (*CHOP*, *PERK*) in Panc1 cells treated as described in (**C**) Quantification of ATF6 activity in Panc1 cells transiently expressing an ATF6 luciferase reporter, treated as described in (**C**) (mean of n=3 ± SD). (**K**) Rel. mRNA expression of *HERPUD1*, an ER-stress gene selective for the ATF6 pathway (Panc1 cells treated as in (**B**); mean of n=3 ± SD)

Next, we aimed to clarify further the effect of ISX/Gem on ER-stress in cancer cells. To this end, we analyzed the three individual branches of the ER-resident unfolded protein response (UPR) system [21, 22]: The IRE1, PERK, and ATF6 arms. First, we investigated the IRE1 branch using a fluorescent reporter [23] (Fig. 2A). While the exposure of cells to ISX or Gem alone evoked no or only moderate responses, the combination of ISX/Gem led to pronounced activation of IRE1 signaling, similar to the positive control Thapsigargin (Fig. 2B,C and Fig. S2 A). Comparable data were obtained using the approved HDACi Chidamide and MS- 275, which demonstrated that this effect is not unique to ISX (Fig. S2B,C,D). In agreement, the IRE1-mediated splice product *XBP1 s* as well as its target gene *DnaJ heat shock protein family (Hsp40) member B9* (*DNAJB*9) [24] were also significantly increased upon HDACi/Gem treatment (Fig. 2D and Fig. S2E).

In order to study the next UPR branch, we used a fluorescent reporter to measure the activity of the PERK axis [23] (Fig. 2E). Again, the ISX/Gem combination resulted in significant induction of this ER-stress pathway as well (Fig. 2F,G and Fig. S2 F), as did the combination of Gem with established HDACi (Fig. S2G,H,I). In support of these findings, the Activating transcription factor 4 (ATF4) target genes *PERK* and *CHOP* were also induced on mRNA level (Fig. 2H and S2 J).

Finally, we investigated ATF6 activation by means of a luminometric reporter harboring ATF6-binding sites [25] (Fig. 2I). Similar to our previous data, individual treatment had a marginal impact, but the combination of ISX/Gem significantly induced the ATF6 ER-stress pathway. However, in this particular ER-stress branch, the combination treatment had a weaker effect compared to the positive control, revealing a certain degree of pathway selectivity (Fig. 2 J). In support of these findings, the ATF6 target gene *Homocysteine inducible ER protein with ubiquitin like domain 1* (*HERPUD1*) was found to be clearly induced upon double treatment but less than the positive control Thapsigargin (Fig. 2 K). These data were confirmed using Chidamide and MS- 275 as clinical HDACi (Fig. S2 K,L). Furthermore, we could show in another cell line (MIA PaCa- 2), that the combination of ISX/Gem synergistically induced *CHOP* gene expression (Fig. S2M). Taken together, we found that HDACi synergize with Gem in eliciting ER-stress and activating all three major UPR branches in PDAC cancer cells, with a preference for the PERK/ATF4 and IRE1/XBP1 arms and with a weaker activation of the ATF6 arm.

### ER-phagy precedes ER-stress and results in apoptosis

**Fig. 3:**
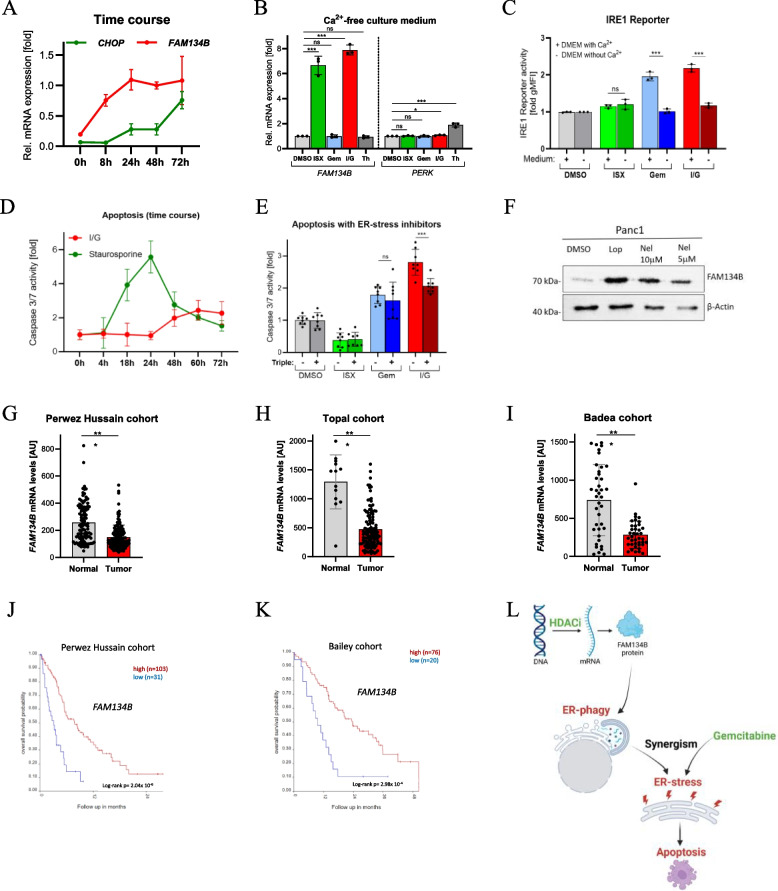
The impact of HDACi/Gem on apoptosis and its link to clinical data. (**A**) Rel. mRNA expression of *FAM134B* and *CHOP* (mean of n=3 ± SD). Panc1 cells were treated with DMSO or the combination of 20 µM ISX and 1.0 µM Gem for different time periods. (**B**) Rel. mRNA expression of *FAM134B* and *PERK* (mean of n=3 ± SD). Panc1 cells were treated with DMSO, 20 µM ISX, 1.0 µM Gem, dual treatment (I/G), or 1.0 µM Thapsigargin (Th) in calcium-free or calcium-containing DMEM medium for 48h. (**C**) Flow cytometric quantification of IRE1 activity in Panc1 cells stably expressing an IRE1 reporter construct. Treatment as in (**B**) (n=3 ± SD). (**D**) Caspase 3/7 activity in Panc1 cells exposed to ISX (I, 20 µM) and Gem (G, 1.0 µM) for various time periods. Staurosporine (1.0 µM) was included as a positive control. The mean ± SD of two representative experiments measured in triplicates is shown. (**E**) Caspase 3/7 activity in Panc1 cells treated with the indicated drugs for 60h. ISX (I, 20 µM); Gem (G, 1.0 µM); IRE1-inh. 4µ8c (30µM); PERK-inh. ISRIB (1.0µM); ATF6-inh. Ceapin-A7 (6.0µM). Shown is the mean (± SD) of two representative experiments measured in quadruplicate each. Each dot represents one biological sample. (**F**) Western blot analysis of FAM134B protein levels in Panc1 cells treated for 48h with DMSO, 20 µM Loperamide, or 5/10 µM Nelfinavir. Shown is one representative experiment of n=5. β-Actin was used as a loading control. (**G**, **H**, **I**) *FAM134B* mRNA expression in bulk tissue from PDAC patients and corresponding healthy pancreatic tissue. Each dot represents one patient. (**J**, **K**) Kaplan-Meier plots representing the overall survival of patients with PDAC as a function of *FAM134B* expression (Scan-split; log-rank p). (**L**) Scheme illustrating the ER-phagy-induced mechanism by which HDACi and Gemcitabine synergize in their anti-tumor effectiveness

The intertwined relationship between ER-stress and ER-phagy prompted us to inquire about the temporal sequence of events. To this end, we used *FAM134B* and *CHOP* gene expression as surrogate read-outs for ER-phagy and ER-stress, respectively. Interestingly, ISX/Gem quickly induced *FAM134B* expression (within hours) while *CHOP* levels were increased with a delay (within days). This indicates that ER-phagy is the initial event which is followed by ER-stress (Fig. 3A). Given that these two events could be separated on a temporal scale, we investigated the role of extracellular calcium as Ca^2+^ is a key contributor to proper protein folding within the ER and thus to the UPR. Culturing PDAC cancer cells in calcium-free medium had no effect on the ability of ISX to induce *FAM134B* mRNA expression (Fig. 3B). In contrast, *PERK* induction (as a transcriptional measure for ER-stress) was almost completely lost under these experimental conditions (Fig. 3B; compare to Fig. 2H), as was the activity of the IRE1 reporter (Fig. 3C and Fig. S3 A). These data imply that ER-phagy and ER-stress are temporally and mechanistically independent events induced by ISX/Gem.

In light of literature that describes a connection between elevated *CHOP* gene expression and apoptosis [26], we measured the activity of the pro-apoptotic caspases 3/7. This approach was supported by previous data on the cytotoxicity of HDACi/Gem treatment in PDAC cells [6, 7]. In line with published data, ISX/Gem significantly increased apoptotic rates in Panc1 cells (Fig. S3B). However, this effect required at least 48 h of treatment, arguing that it kinetically followed after the sequence of ER-phagy/ER-stress and was likely the result of unresolved ER-stress (Fig. 3D; compare to Fig. 3A).

To prove our hypothesis that the sequence of ER-phagy/ER-stress causes apoptosis, we applied selective inhibitors for the IRE1-, PERK-, and ATF6-branches (i.e. 4µ8C, ISRIB, Ceapin-A7, respectively) to ISX/Gem-treated cells and quantified apoptotic rates. As shown in figure S3 C, the application of inhibitors individually had only a limited impact*,* with inhibition of the IRE1 arm having the largest effect. In contrast, the addition of all three inhibitors simultaneously (‘triple inhibition’) completely abrogated the synergistic effect of ISX/Gem double treatment on apoptosis and brought apoptotic levels back to ‘Gem-only’ conditions (Fig. 3E). These results demonstrated that all three branches of the UPR contributed to the synergistic impact of cell killing. ISX/Gem-induced apoptosis was additionally quantified by Annexin-V/PI-staining (using flow cytometry) (Fig. S3D,E) and was also verified using approved HDACi (Fig. S3 F).

Intriguingly, we could show that HDACi-unrelated compounds like Loperamide, a µ-opioid receptor agonist, and Nelfinavir, used for the treatment of acquired immunodeficiency syndrome, can also cause FAM134B upregulation (Fig. 3F and Fig. S3G). Because these substances can thus potentially initiate the ‘FAM134B →ER-phagy→ER-stress→apoptosis’ axis, they might expand the range of possible Gem-synergizing compounds.

Eventually, after having provided experimental evidence for a series of events initially triggered by an HDACi-induced *FAM134B* upregulation, we wondered about *FAM134B* expression in human PDAC patients, which typically receive adjuvant or neoadjuvant chemotherapy often containing Gemcitabine. Interestingly, when investigating three separate publicly available bulk transcriptomic pancreatic cancer data sets, including also normal pancreatic tissue as controls [27–29], we detected significantly decreased levels of *FAM134B* gene expression in tumors versus healthy controls (Fig. 3G,H,I). These data imply a general anti-tumoral impact of this gene and possibly also of ER-phagy, already in the absence of HDACi treatment. In support of this view, a statistically significant correlation between higher *FAM134B* expression and improved overall survival could also be verified in three large public data sets [29, 30] (Fig. 3 J,K and Fig. S3H).

In conclusion, we found that HDAC inhibition results in the upregulation of *FAM134B* and the induction of ER-phagy. When combined with Gemcitabine, this is followed by the synergistic elevation of ER-stress levels within days, which induces apoptosis. Thus, the sequence of ER-phagy/ER-stress is absolutely critical for cell killing as the inhibition of the ER-stress effectors abrogates apoptosis synergism (Fig. 3L). Compared to healthy tissue, *FAM134B* mRNA levels are decreased in pancreatic tumors, and the expression level positively correlates with overall patient survival.

## Discussion

Compelling evidence in the literature demonstrates the synergistic effect of HDAC inhibition with chemotherapy. Here, we could surprisingly find that ER-phagy is a central step in this process, emphasizing the role of autophagy as a therapeutic target [31]. In normal physiology, ER-phagy contributes to ER quality control and homeostasis as well as to innate immune signaling [32–34]. At the same time, mutations in ER-phagy receptors have been associated with certain diseases [35]. Our data provide evidence that HDACi increase ER-phagy mainly through *FAM134B* expression, which subsequently triggers ER-stress. On the other hand, Gemcitabine has also been shown to elicit ER-stress [36], potentially explaining why these two compounds perfectly synergize on a functional level, and supporting previous data on ER-stress impacting chemosensitivity in cancer cells [37, 38]. These two events are closely interconnected and can reinforce each other: ER-stress can result from ER-phagy [39, 40], but ER-phagy can also be induced as a compensatory mechanism after ER-stress to restore homeostasis [41].

Intriguingly, completely HDACi-unrelated small molecules (e.g. Loperamide, Nelfinavir) can also enter this ‘FAM134B→ ER-phagy→ER-stress→apoptosis’ axis, potentially explaining the mechanism of promising clinical results obtained with Nelfinavir plus chemoradiation in locally advanced PDAC and of Loperamide in glioma cells [42, 43]. This knowledge might open the possibility for future identification of novel drugs to use autophagy, in this case selective ER-phagy induction, to increase chemosensitivity in cancer patients.

In general, HDAC inhibitors have long been known to promote cell differentiation [44], a feature that was recently also associated with ER-phagy in the process of neuronal differentiation [45]. Notably, ISX was originally identified as a pro-neurogenic compound [17], and it can be proposed that the promotion of ER-phagy might as well contribute to this feature.

In addition, and highly relevant from a clinical viewpoint, we found that basal *FAM134B* levels (i.e. without parallel HDACi therapy) were already significantly correlated with overall survival in PDAC patients. This could suggest that ER-phagy as a whole might serve general tumor-suppressive functions. In HDACi treatment settings, these anti-tumoral features are amplified through an HDACi-induced *FAM134B* increase. In this respect, it is surprising to note that HDACi, unfortunately, have not fully convinced in clinical trials of solid cancer so far, with both promising [46, 47] as well as disappointing [48, 49] results being reported. In many instances, HDACi treatment was associated with undesired side effects, often limiting the maximally tolerated dose in patients and resulting in the discontinuation of trials. Novel compounds with indirect mechanisms, such as the non-canonical HDACi ISX, or re-purposed drugs inducing ER-phagy, might represent interesting candidates for further development because of less side effects and the possibility to reach higher dosages over prolonged time.

In conclusion, we could demonstrate that FAM134B-induced ER-phagy plays a key role in the functional interaction of HDACi and Gemcitabine. We were able to provide a mechanistic framework for this clinically relevant synergism, which might aid in a more profound understanding of this therapeutic approach and lead to the development of novel compounds targeting these processes.

## Supplementary Information

Supplementary Material 1

## Data Availability

No datasets were generated or analysed during the current study.

## Abbreviations

ATF6: Activating transcription factor 6
ER: Endoplasmic reticulum
UPR: ER-resident unfolded protein response
FAM134B: Family with sequence similarity 134, member B
Gem: Gemcitabine
HDACi: HDAC inhibitor
IRE1: Inositol-requiring enzyme 1
ISX: Isoxazole 9
PDAC: Pancreatic ductal adenocarcinoma
PI: Propidium iodide
RT: Room temperature
TEM: Transmission electron microscopy
PERK: Protein kinase R (PKR)-like ER kinase

## References

[1] Chen I-C, Sethy B, Liou J-P. Recent Update of HDAC Inhibitors in Lymphoma. Frontiers in cell and developmental biology. 2020;8: 576391. 33015069 10.3389/fcell.2020.576391 PMC7494784

[2] Irimia R, Piccaluga PP. Histone Deacetylase Inhibitors for Peripheral T-Cell Lymphomas. Cancers. 2024;16:3359. 10.3390/cancers16193359 PMC11482620 39409979

[3] Mabe NW, Perry JA, Malone CF, Stegmaier K. Pharmacological targeting of the cancer epigenome. Nature cancer. 2024;5:844–65. 38937652 10.1038/s43018-024-00777-2 PMC11936478

[4] Falkenberg KJ, Johnstone RW. Histone deacetylases and their inhibitors in cancer, neurological diseases and immune disorders. Nat Rev Drug Discovery. 2014;13:673–91. 25131830 10.1038/nrd4360

[5] Knoche SM, Brumfield GL, Goetz BT, Sliker BH, Larson AC, Olson MT, Poelaert BJ, Bavari A, Yan Y, Black JD, Solheim JC. The histone deacetylase inhibitor M344 as a multifaceted therapy for pancreatic cancer. PLoS ONE. 2022;17: e0273518. 36126055 10.1371/journal.pone.0273518 PMC9488834

[6] Maietta I, Martínez-Pérez A, Álvarez R, de Lera ÁR, González-Fernández Á, Simón-Vázquez R. Synergistic Antitumoral Effect of Epigenetic Inhibitors and Gemcitabine in Pancreatic Cancer Cells. Pharmaceuticals: Basel, Switzerland; 2022:15. 10.3390/ph15070824 PMC9323654 35890123

[7] Gahr S, Ocker M, Ganslmayer M, Zopf S, Okamoto K, Hartl A, Leitner S, Hahn EG, Herold C. The combination of the histone-deacetylase inhibitor trichostatin A and gemcitabine induces inhibition of proliferation and increased apoptosis in pancreatic carcinoma cells. Int J Oncol. 2007;31:567–76. 17671683

[8] Laschanzky RS, Humphrey LE, Ma J, Smith LM, Enke TJ, Shukla SK, Dasgupta A, Singh PK, Howell GM, Brattain MG, Ly QP, Black AR, Black JD. Selective Inhibition of Histone Deacetylases 1/2/6 in Combination with Gemcitabine: A Promising Combination for Pancreatic Cancer Therapy. Cancers. 2019;11:1327. 10.3390/cancers11091327 PMC6770665 31500290

[9] Qiao Z, Ren S, Li W, Wang X, He M, Guo Y, Sun L, He Y, Ge Y, Yu Q. Chidamide, a novel histone deacetylase inhibitor, synergistically enhances gemcitabine cytotoxicity in pancreatic cancer cells. Biochem Biophys Res Commun. 2013;434:95–101. 23541946 10.1016/j.bbrc.2013.03.059

[10] Schober M, Jesenofsky R, Faissner R, Weidenauer C, Hagmann W, Michl P, Heuchel RL, Haas SL, Löhr J-M. Desmoplasia and chemoresistance in pancreatic cancer. Cancers. 2014;6:2137–54. 25337831 10.3390/cancers6042137 PMC4276960

[11] Halbrook CJ, Thurston G, Boyer S, Anaraki C, Jiménez JA, McCarthy A, Steele NG, Kerk SA, Hong HS, Lin L, Law FV, Felton C, Scipioni L, Sajjakulnukit P, Andren A, Beutel AK, Singh R, Nelson BS, van den Bergh F, Krall AS, Mullen PJ, Zhang L, Batra S, Morton JP, Stanger BZ, Christofk HR, Digman MA, Beard DA, Viale A, Zhang J, et al. Differential integrated stress response and asparagine production drive symbiosis and therapy resistance of pancreatic adenocarcinoma cells. Nature cancer. 2022;3:1386–403. 36411320 10.1038/s43018-022-00463-1 PMC9701142

[12] Koeniger A, Brichkina A, Nee I, Dempwolff L, Hupfer A, Galperin I, Finkernagel F, Nist A, Stiewe T, Adhikary T, Diederich W, Lauth M. Activation of Cilia-Independent Hedgehog/GLI1 Signaling as a Novel Concept for Neuroblastoma Therapy. Cancers 2021;13:1908. 10.3390/cancers13081908 PMC8071409 33921042

[13] Koeniger A, Polo P, Brichkina A, Finkernagel F, Visekruna A, Nist A, Stiewe T, Daude M, Diederich WE, Gress TM, Adhikary T, Lauth M. Tumor-suppressive disruption of cancer subtype-associated super enhancer circuits by small molecule treatment. NAR cancer. 2023;5:zcad007. 10.1093/narcan/zcad007 PMC9900422 36755960

[14] Hupfer A, Brichkina A, Adhikary T, Lauth M. The mammalian Hedgehog pathway is modulated by ANP32 proteins. Biochem Biophys Res Commun. 2021;553:78–84. 33761414 10.1016/j.bbrc.2021.03.027

[15] Helmprobst F, Frank M, Stigloher C. Presynaptic architecture of the larval zebrafish neuromuscular junction. J Comp Neurol. 2015;523:1984–97. 25766140 10.1002/cne.23775

[16] Kremer JR, Mastronarde DN, McIntosh JR. Computer visualization of three-dimensional image data using IMOD. J Struct Biol. 1996;116:71–6. 8742726 10.1006/jsbi.1996.0013

[17] Schneider JW, Gao Z, Li S, Farooqi M, Tang T-S, Bezprozvanny I, Frantz DE, Hsieh J. Small-molecule activation of neuronal cell fate. Nat Chem Biol. 2008;4:408–10. 18552832 10.1038/nchembio.95

[18] Reggiori F, Molinari M. ER-phagy: mechanisms, regulation, and diseases connected to the lysosomal clearance of the endoplasmic reticulum. Physiol Rev. 2022;102:1393–448. 35188422 10.1152/physrev.00038.2021 PMC9126229

[19] Gubas A, Dikic I. ER remodeling via ER-phagy. Mol Cell. 2022;82:1492–500. 35452617 10.1016/j.molcel.2022.02.018 PMC9098120

[20] Stefely JA, Zhang Y, Freiberger EC, Kwiecien NW, Thomas HE, Davis AM, Lowry ND, Vincent CE, Shishkova E, Clark NA, Medvedovic M, Coon JJ, Pagliarini DJ, Mercer CA. Mass spectrometry proteomics reveals a function for mammalian CALCOCO1 in MTOR-regulated selective autophagy. Autophagy. 2020;16:2219–37. 31971854 10.1080/15548627.2020.1719746 PMC7751563

[21] Chen X, Cubillos-Ruiz JR. Endoplasmic reticulum stress signals in the tumour and its microenvironment. Nat Rev Cancer. 2021;21:71–88. 33214692 10.1038/s41568-020-00312-2 PMC7927882

[22] Pakos-Zebrucka K, Koryga I, Mnich K, Ljujic M, Samali A, Gorman AM. The integrated stress response. EMBO Rep. 2016;17:1374–95. 27629041 10.15252/embr.201642195 PMC5048378

[23] Nougarède A, Tesnière C, Ylanko J, Rimokh R, Gillet G, Andrews DW. Improved IRE1 and PERK Pathway Sensors for Multiplex Endoplasmic Reticulum Stress Assay Reveal Stress Response to Nuclear Dyes Used for Image Segmentation. Assay Drug Dev Technol. 2018;16:350–60. 30088945 10.1089/adt.2018.862

[24] Grandjean JMD, Madhavan A, Cech L, Seguinot BO, Paxman RJ, Smith E, Scampavia L, Powers ET, Cooley CB, Plate L, Spicer TP, Kelly JW, Wiseman RL. Pharmacologic IRE1/XBP1s activation confers targeted ER proteostasis reprogramming. Nat Chem Biol. 2020;16:1052–61. 32690944 10.1038/s41589-020-0584-z PMC7502540

[25] Wang Y, Shen J, Arenzana N, Tirasophon W, Kaufman RJ, Prywes R. Activation of ATF6 and an ATF6 DNA binding site by the endoplasmic reticulum stress response. J Biol Chem. 2000;275:27013–20. 10856300 10.1074/jbc.M003322200

[26] Hu H, Tian M, Ding C, Yu S. The C/EBP Homologous Protein (CHOP) Transcription Factor Functions in Endoplasmic Reticulum Stress-Induced Apoptosis and Microbial Infection. Front Immunol. 2018;9:3083. 30662442 10.3389/fimmu.2018.03083 PMC6328441

[27] Janky R, Binda MM, Allemeersch J, van den Broeck A, Govaere O, Swinnen JV, Roskams T, Aerts S, Topal B. Prognostic relevance of molecular subtypes and master regulators in pancreatic ductal adenocarcinoma. BMC Cancer. 2016;16:632. 27520560 10.1186/s12885-016-2540-6 PMC4983037

[28] Badea L, Herlea V, Dima SO, Dumitrascu T, Popescu I. Combined gene expression analysis of whole-tissue and microdissected pancreatic ductal adenocarcinoma identifies genes specifically overexpressed in tumor epithelia. Hepatogastroenterology. 2008;55:2016–27. 19260470

[29] Yang S, Tang W, Azizian A, Gaedcke J, Ströbel P, Wang L, Cawley H, Ohara Y, Valenzuela P, Zhang L, Lal T, Sinha S, Rupin E, Hanna N, Ghadimi BM, Hussain SP. Dysregulation of HNF1B/Clusterin axis enhances disease progression in a highly aggressive subset of pancreatic cancer patients. Carcinogenesis. 2022;43:1198–210. 36426859 10.1093/carcin/bgac092 PMC10122429

[30] Bailey P, Chang DK, Nones K, Johns AL, Patch A-M, Gingras M-C, Miller DK, Christ AN, Bruxner TJC, Quinn MC, Nourse C, Murtaugh LC, Harliwong I, Idrisoglu S, Manning S, Nourbakhsh E, Wani S, Fink L, Holmes O, Chin V, Anderson MJ, Kazakoff S, Leonard C, Newell F, Waddell N, Wood S, Xu Q, Wilson PJ, Cloonan N, Kassahn KS, et al. Genomic analyses identify molecular subtypes of pancreatic cancer. Nature. 2016;531:47–52. 26909576 10.1038/nature16965

[31] Liu H, He Z, Simon H-U. Targeting autophagy as a potential therapeutic approach for melanoma therapy. Semin Cancer Biol. 2013;23:352–60. 23831275 10.1016/j.semcancer.2013.06.008

[32] Wilkinson S. ER-phagy: shaping up and destressing the endoplasmic reticulum. FEBS J. 2019;286:2645–63. 31116513 10.1111/febs.14932 PMC6772018

[33] Grumati P, Dikic I, Stolz A. ER-phagy at a glance. Journal of cell science. 2018;131. 10.1242/jcs.217364 30177506

[34] Dikic I. Open questions: why should we care about ER-phagy and ER remodelling? BMC Biol. 2018;16:131. 30382915 10.1186/s12915-018-0603-7 PMC6211458

[35] Hübner CA, Dikic I. ER-phagy and human diseases. Cell Death Differ. 2020;27:833–42. 31659280 10.1038/s41418-019-0444-0 PMC7206075

[36] Wang L, Zhang Y, Wang W, Zhu Y, Chen Y, Tian B. Gemcitabine treatment induces endoplasmic reticular (ER) stress and subsequently upregulates urokinase plasminogen activator (uPA) to block mitochondrial-dependent apoptosis in Panc-1 cancer stem-like cells (CSCs). PLoS ONE. 2017;12: e0184110. 28854261 10.1371/journal.pone.0184110 PMC5576696

[37] Gilabert M, Vaccaro MI, Fernandez-Zapico ME, Calvo EL, Turrini O, Secq V, Garcia S, Moutardier V, Lomberk G, Dusetti N, Urrutia R, Iovanna JL. Novel role of VMP1 as modifier of the pancreatic tumor cell response to chemotherapeutic drugs. J Cell Physiol. 2013;228:1834–43. 23460482 10.1002/jcp.24343 PMC4048029

[38] Kutschat AP, Hamdan FH, Wang X, Wixom AQ, Najafova Z, Gibhardt CS, Kopp W, Gaedcke J, Ströbel P, Ellenrieder V, Bogeski I, Hessmann E, Johnsen SA. STIM1 Mediates Calcium-Dependent Epigenetic Reprogramming in Pancreatic Cancer. Can Res. 2021;81:2943–55. 10.1158/0008-5472.CAN-20-2874 PMC8178177 33436389

[39] Liao Y, Duan B, Zhang Y, Zhang X, Xia B. Excessive ER-phagy mediated by the autophagy receptor FAM134B results in ER stress, the unfolded protein response, and cell death in HeLa cells. J Biol Chem. 2019;294:20009–23. 31748416 10.1074/jbc.RA119.008709 PMC6937584

[40] Cinque L, de Leonibus C, Iavazzo M, Krahmer N, Intartaglia D, Salierno FG, de Cegli R, Di Malta C, Svelto M, Lanzara C, Maddaluno M, Wanderlingh LG, Huebner AK, Cesana M, Bonn F, Polishchuk E, Hübner CA, Conte I, Dikic I, Mann M, Ballabio A, Sacco F, Grumati P, Settembre C. MiT/TFE factors control ER-phagy via transcriptional regulation of FAM134B. EMBO J. 2020;39: e105696. 32716134 10.15252/embj.2020105696 PMC7459426

[41] Prieto-Garcia C, Matkovic V, Mosler T, Li C, Liang J, Oo JA, Haidle F, Mačinković I, Cabrera-Orefice A, Berkane R, Giuliani G, Xu F, Jacomin A-C, Tomaskovic I, Basoglu M, Hoffmann ME, Rathore R, Cetin R, Boutguetait D, Bozkurt S, Hernández Cañás MC, Keller M, Busam J, Shah VJ, Wittig I, Kaulich M, Beli P, Galej WP, Ebersberger I, Wang L *et al..* Pathogenic proteotoxicity of cryptic splicing is alleviated by ubiquitination and ER-phagy. Science (New York, N.Y.). 2024;386:768–776. 10.1126/science.adi5295 39541449

[42] Wilson JM, Fokas E, Dutton SJ, Patel N, Hawkins MA, Eccles C, Chu K-Y, Durrant L, Abraham AG, Partridge M, Woodward M, O’Neill E, Maughan T, McKenna WG, Mukherjee S, Brunner TB. ARCII: A phase II trial of the HIV protease inhibitor Nelfinavir in combination with chemoradiation for locally advanced inoperable pancreatic cancer. Radiotherapy and oncology: journal of the European Society for Therapeutic Radiology and Oncology. 2016;119:306–11. 27117177 10.1016/j.radonc.2016.03.021 PMC4917892

[43] Zielke S, Kardo S, Zein L, Mari M, Covarrubias-Pinto A, Kinzler MN, Meyer N, Stolz A, Fulda S, Reggiori F, Kögel D, van Wijk S. ATF4 links ER stress with reticulophagy in glioblastoma cells. Autophagy. 2021;17:2432–48. 33111629 10.1080/15548627.2020.1827780 PMC8496713

[44] Leder A, Leder P. Butyric acid, a potent inducer of erythroid differentiation in cultured erythroleukemic cells. Cell. 1975;5:319–22. 1056809 10.1016/0092-8674(75)90107-5

[45] Hoyer MJ, Capitanio C, Smith IR, Paoli JC, Bieber A, Jiang Y, Paulo JA, Gonzalez-Lozano MA, Baumeister W, Wilfling F, Schulman BA, Harper JW. Combinatorial selective ER-phagy remodels the ER during neurogenesis. Nat Cell Biol. 2024;26:378–92. 38429475 10.1038/s41556-024-01356-4 PMC10940164

[46] Ikeda M, Ohno I, Ueno H, Mitsunaga S, Hashimoto Y, Okusaka T, Kondo S, Sasaki M, Sakamoto Y, Takahashi H, Hara R, Kobayashi S, Nakamura O, Morizane C. Phase I study of resminostat, an HDAC inhibitor, combined with S-1 in patients with pre-treated biliary tract or pancreatic cancer. Invest New Drugs. 2019;37:109–17. 29995287 10.1007/s10637-018-0634-5

[47] Jo JH, Jung DE, Lee HS, Park SB, Chung MJ, Park JY, Bang S, Park SW, Cho S, Song SY. A phase I/II study of ivaltinostat combined with gemcitabine and erlotinib in patients with untreated locally advanced or metastatic pancreatic adenocarcinoma. Int J Cancer. 2022;151:1565–77. 35657348 10.1002/ijc.34144 PMC9545559

[48] Chan E, Chiorean EG, O’Dwyer PJ, Gabrail NY, Alcindor T, Potvin D, Chao R, Hurwitz H. Phase I/II study of mocetinostat in combination with gemcitabine for patients with advanced pancreatic cancer and other advanced solid tumors. Cancer Chemother Pharmacol. 2018;81:355–64. 29238851 10.1007/s00280-017-3494-3

[49] Wang H, Cao Q, Dudek AZ. Phase II study of panobinostat and bortezomib in patients with pancreatic cancer progressing on gemcitabine-based therapy. Anticancer Res. 2012;32:1027–31. 22399627

