# Supplementary material for: ER-phagy mediates the anti-tumoral synergism between HDAC inhibition and chemotherapy
Source: Cell Commun Signal. 2025 Apr 26;23:202. doi: 10.1186/s12964-025-02198-9 (PMC12034116; doi:10.1186/s12964-025-02198-9)

Supplemental Information

ER-phagy mediates the anti-tumoral synergism between HDAC inhibition and chemotherapy

Felix J. Gössl, Pierfrancesco Polo, Frederik Helmprobst, André Menzenbach, Alexander Visekruna, Thomas M. Gress, Till Adhikary, Matthias Lauth.

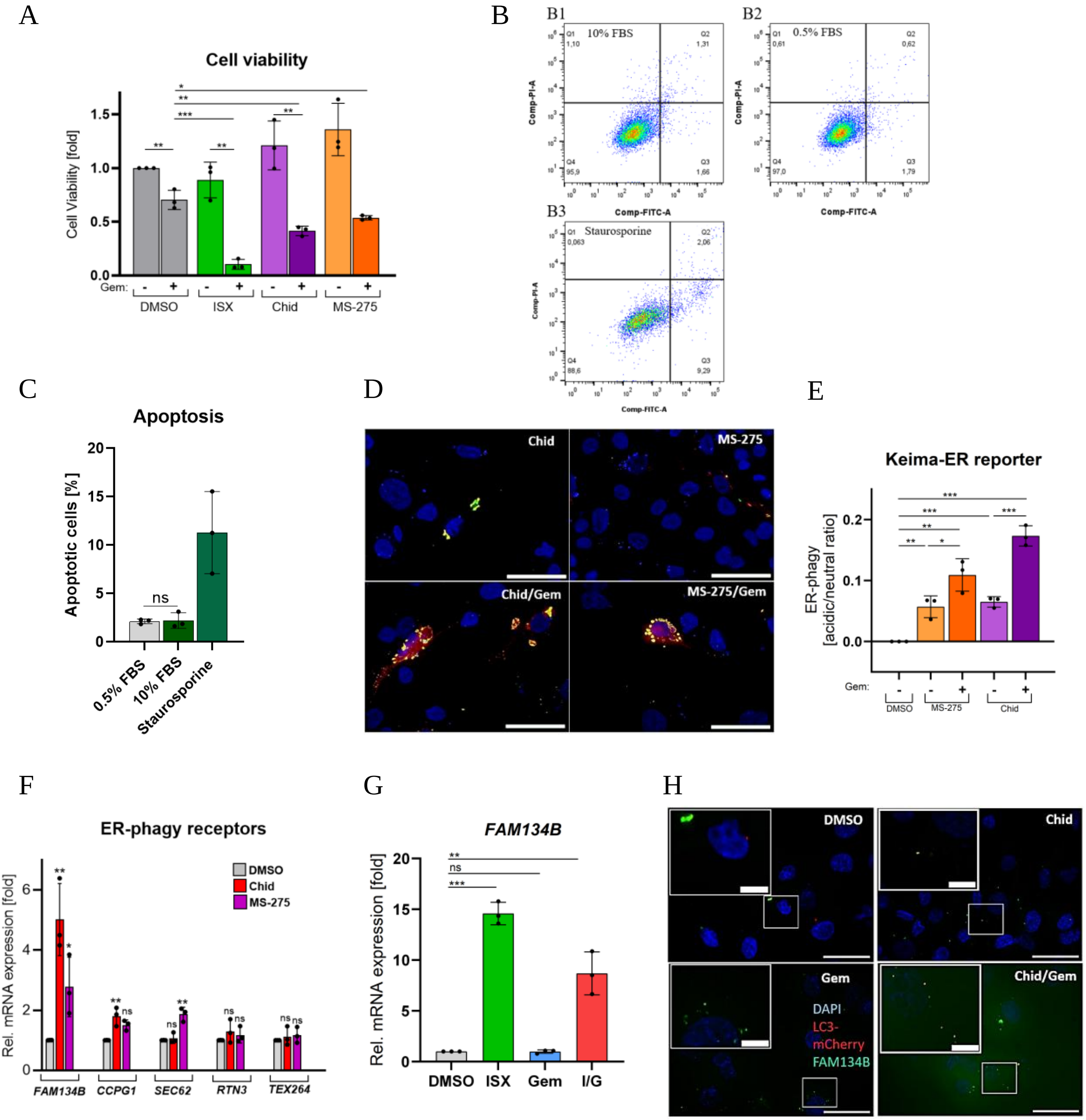

Figure S1. ISX induces ER-phagy. (**A**) Cell viability assay: Panc1 cells were treated with DMSO, 20 µM ISX, 1.0 µM Gem, 5.0 µM Chidamide (Chid) and 1.0 µM MS-275 for 4 days (mean of n=3 ± SD). (**B**) Dot plot showing Panc1 cells treated with medium containg 0.5% FBS, 10% FBS or 1.0 µM Staurosporine in 0.5% FBS for 60h, stained with Annexin V-FITC and Propidium Iodide (PI), and analyzed by flow cytometry. Early apoptotic cells appear in Q3 (Annexin V+/PI-), while late apoptotic cells are located in Q2 (Annexin V+/PI+). Shown are the percentages of each quadrant of the overall cell poluation. (**C**) Quantification of apoptosis using Annexin V/PI staining: Cells were treated as described in (B). The percentage of apoptotic cells (Q2, Q3) in the overall cell population is shown (mean of n=3 ± SD). (**D**) Immunofluorescence pictures of Panc1 cells transiently expressing ER-Keima reporter and treated for 48h as described in (A). Scale bar: 60 µm. Shown is one representative experiment of n=2. (**E**) Quantification of ER-phagy using Panc1 cells stably expressing the ER-Keima reporter. These cells were treated as described in (D) and analyzed using flow cytometry (mean of n=3 ± SD). (**F**) Relative mRNA expression of ER-phagy receptors (*FAM134B*, *CCPG1*, *SEC62*, *RTN3*, *TEX264*) in Panc1 cells, treated with DMSO, 5.0 µM Chidamide, 1.0 µM MS-275 for 48h. Shown is the mean ± SD (n=3; one-tailed t-test). (**G**) Relative mRNA expression of *FAM134B* in MIA PaCa-2 cells, treated as described in (D) for 48h (mean of n=3 ± SD). (**H**) Colocalization of transiently transfected Panc1 cells mCherry-LC3B (red) and endogenous FAM134B (green). Treatment as in (D). One representative of n=4 is shown. Scale bar: 50µm in the larger panel and 10µm in the magnified insets.

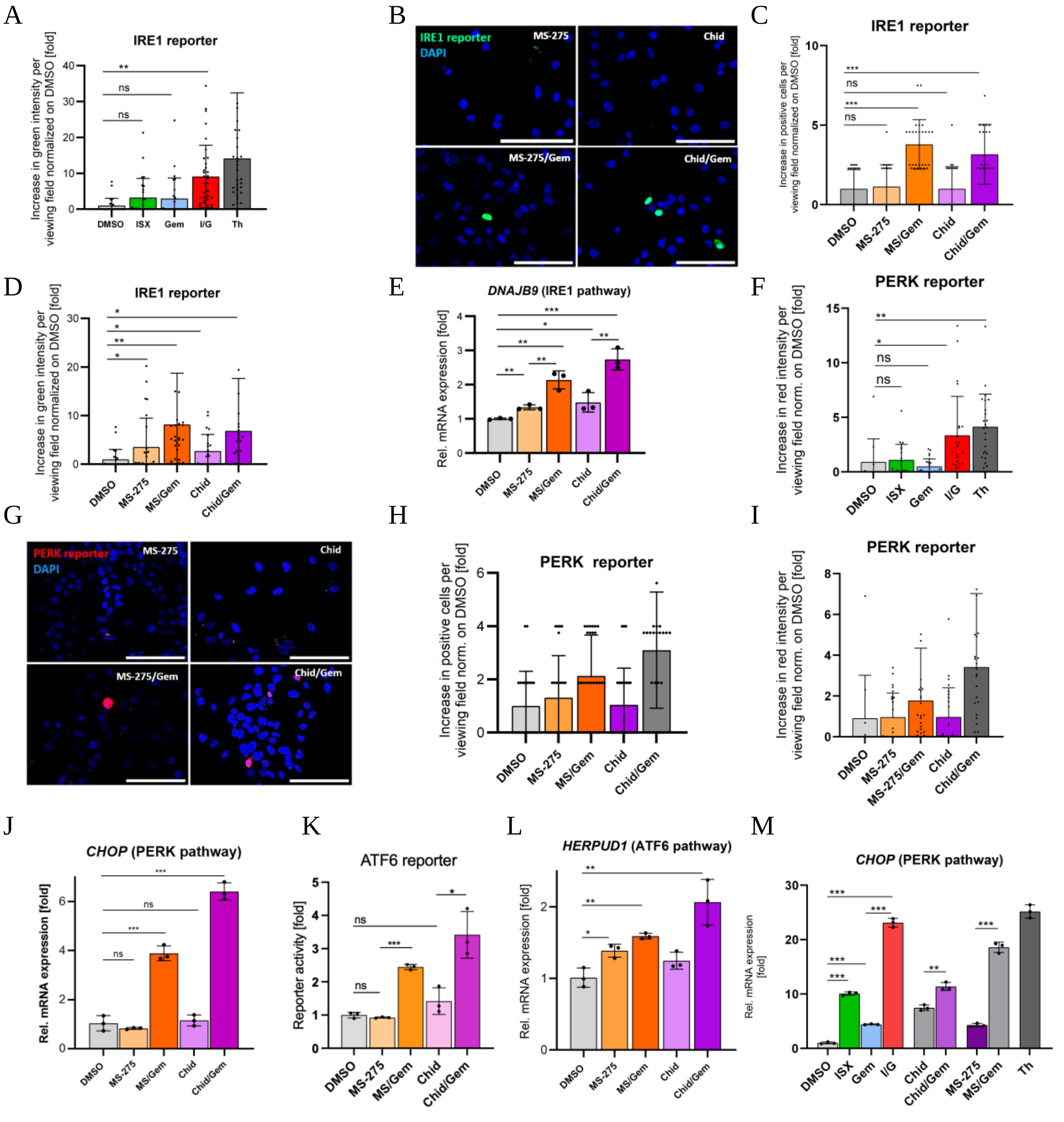

Figure S2. ISX and Gemcitabine synergistically induce ER-stress. **(A)** Quantification of Panc1 cells transiently expressing IRE1 reporter. Cells were treated with DMSO, 20 µM ISX, 1 µM Gem and the combination ISX/Gem (I/G) or 1 µM Thapsigargin (Th) for 48h, analyzed using the mean green intensity per viewing field normalized to the values of the DMSO sample. Each dot represents one image (from n=2 independent experiments; mean ± SD). **(B)** Immunofluorescence pictures of Panc1 cells transiently expressing IRE1 reporter (green), treated with DMSO, 5.0 µM Chidamide (Chid), 1.0 µM MS-275 or 1 µM Gem for 48h. Nuclei appear blue (DAPI). Scale bar 50 µm. Shown is one representative experiment of n=3. **(C,D)** Quantification of IRE1 activity in Panc1 cells transiently expressing an IRE1 reporter construct. Cells were treated as outlined in (B). Analyzed was either the number of positive cells per viewing field (C) or the mean green intensity per viewing field (D). All values were normalized on DMSO condition. Each dot represents one microscopic image from n=2 independent experiments (mean ± SD). **(E)** Rel. mRNA expression of *DNAJB9* in Panc1 cells (mean of n=3 ± SD). **(F)** Quantification of PERK activity in Panc1 cells transiently expressing an PERK reporter construct. Cells were treated as outlined in (A). Analyzed was the mean red intensity per viewing field normalized on DMSO conditions. **(G)** Immunofluorescence pictures of Panc1 cells transiently expressing PERK reporter (red) and treated according to (B). Nuclei appear blue (DAPI). Scale bar 100µm. Shown is one representative experiment of n=3. **(H,I)** Quantification of PERK activity in Panc1 cells transiently transfected with PERK reporter. Cells were treated as outlined in (B). Analyzed was either the number of positive cells per viewing field (H) or the mean red intensity per viewing field (I). All values were normalized on DMSO condition. Each dot represents one microscopic image from n=2 independent experiments (mean ± SD). **(J)** Rel. mRNA expression of *CHOP* in Panc1 cells treated as described in (B) (mean of n=3 ± SD.). **(K)** Quantification of ATF6 activity in Panc1 cells transiently expressing the ATF6 luciferase reporter (treated as described in (B)). Shown is one representative experiment of n=3 measured in triplicate each (mean ± SD). **(L)** Rel. mRNA expression of *HERPUD1* in Panc1 cells (treated as in (B); mean of n=3 ± SD). **(M)** Rel. mRNA expression of *CHOP* in MIA PaCa-2 cells treated with DMSO, 20 µM ISX, 1.0 µM Gem, 1.0 µM Thapsigargin (Th), 5.0 µM Chidamide (Chid) and 1.0 µM MS-275 (MS) for 48h. Shown is one representative experiment of n=3 measured in triplicate each (mean ± SD).

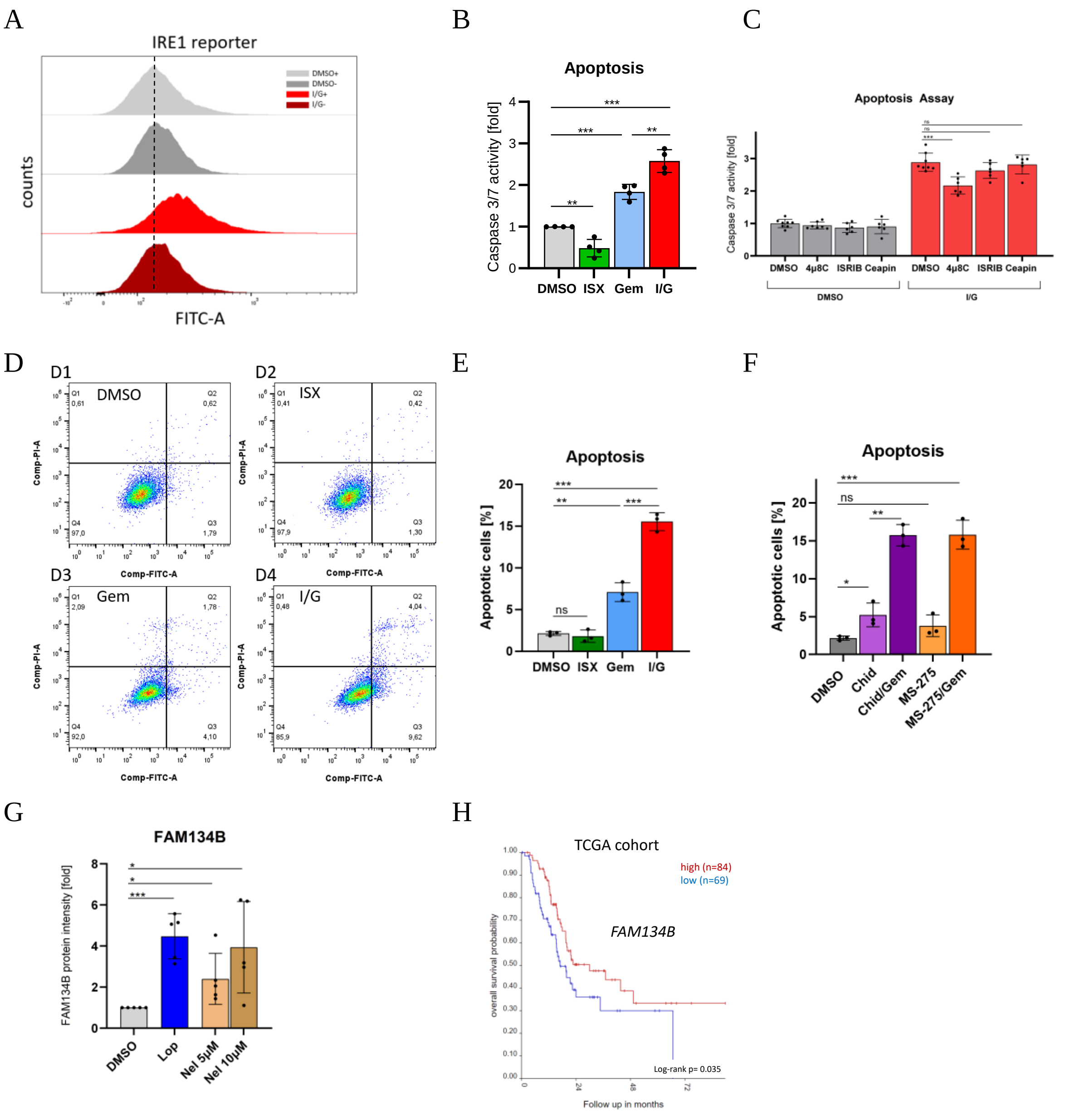

Figure S3. The influence of *FAM134B* expression on apoptosis and its clinical impact. (**A**) Histogram of flow cytometric quantification of IRE1 activity in Panc1 cells stably expressing an IRE1 reporter treated with DMSO or dual treatment of 20 µM ISX, and 1.0 µM Gem (I/G) for 48h in medium containing either Calcium (+) or no Calcium (-). Shown is one representative experiment. **(B)** Caspase 3/7 apoptosis assay in Panc1 cells treated with DMSO, 20 µM ISX, and 1.0 µM Gem or dual treatment (I/G) for 60h (mean of n=4 ± SD). **(C)** Apoptosis assay in Panc1 cells treated as described in (B) and additionally with IRE1-, PERK-, ATF6-Inhibitors (30µM 4µ8c, 1.0µM ISRIB, 6.0µM Ceapin-A7). Shown is the mean ± SD of two independent experiments measured in quadruplicate each. Each dot thus represents one biological sample. (**D**) Dot plot showing Panc1 cells treated as described in (B) for 60h, stained with Annexin V-FITC and Propidium Iodide (PI), and analyzed by flow cytometry. Early apoptotic cells appear in Q3 (Annexin V+/PI-), while late apoptotic cells are located in Q2 (Annexin V+/PI+). Shown are the percentages of each quadrant of the overall cell population. (**E-F**) Quantification of apoptosis using Annexin V/PI staining: Cells were treated with DMSO, 20 µM ISX, 1.0 µM Gem, 5.0 µM Chidamide (Chid), and 1.0 µM MS-275 for 60h. The percentage of apoptotic cells in the overall cell population (Q2, Q3) is shown (mean of n=3 ± SD). **(G)** Quantification of FAM134B protein levels as depicted in (Fig. 3F) (mean of n=5 ± SD). **(H)** Kaplan-Meier plot representing the overall survival of patients with PDAC as a function of *FAM134B* expression (split-scan; log-rank p).

Table 1: qPCR-Primers and corresponding sequences.

Transcript (Hs)	Sequence (5’-3’)
***CCPG1*_for**	CCACGAAGATGAGCTGGATGGT
***CCPG1*_rev**	CAGTAACGGTCCAACACCTCTC
***CHOP* (*DDIT3*)_for**	AGCTGGGAGCTGGAAGCCTGGTATG
***CHOP* (*DDIT3*)_rev**	CCAAGCCAGAGAAGCAGGGTCAAGA
***DNAJB9*_for**	AGTTCAAGGAGATCGCTGAGGC
***DNAJB9*_rev**	GCTGAAAGAGGTACCATTGGCAC
***FAM134B* (*RETREG1*)_for**	GTCTCAGAGGTATCCTGGACTG
***FAM134B* (*RETREG1*)_rev**	TTCCTCACTGGGTCGGTCAAGA
***P0*_qFor**	CCTTCTCCTTTGGGCTGGTCATCCA
***P0*_qRev**	CAGACACTGGCAACATTGCGGACAC
***PERK*_for**	CGAGAGCCGGATTTATTGAAAGCACCT
***PERK*_rev**	TGGCAGCTTCCTGTTCTTCCACATCT
***HERPUD1*_for**	CCAATGTCTCAGGGACTTGCTTC
***HERPUD1*_rev**	CGATTAGAACCAGCAGGCTCCT
***RTN3*_for**	CTTACCTCATCCTGGCTCTTCTC
***RTN3*_rev**	GACAGAGTAATGTCTACGTCCAG
***SEC62*_for**	ACCTCAGTGTGGGTGCAGGCTGTTT
***SEC62*_rev**	GAACCAAAAGTGGTGCCTTCCTCCA
***TEX264*_qFor**	TGCCTGAGATGAAGGAGACAGAGTGGA
***TEX264*_qRev**	TGCCAGGGCTCACTTCCAAGCTTAC
***XBP1s*_for**	TTAAGACAGCGCTTGGGGATGGATG
***XBP1s*_rev**	TGGGCCTGCACCTAATAAGCGGACTC

Western-Blot originals

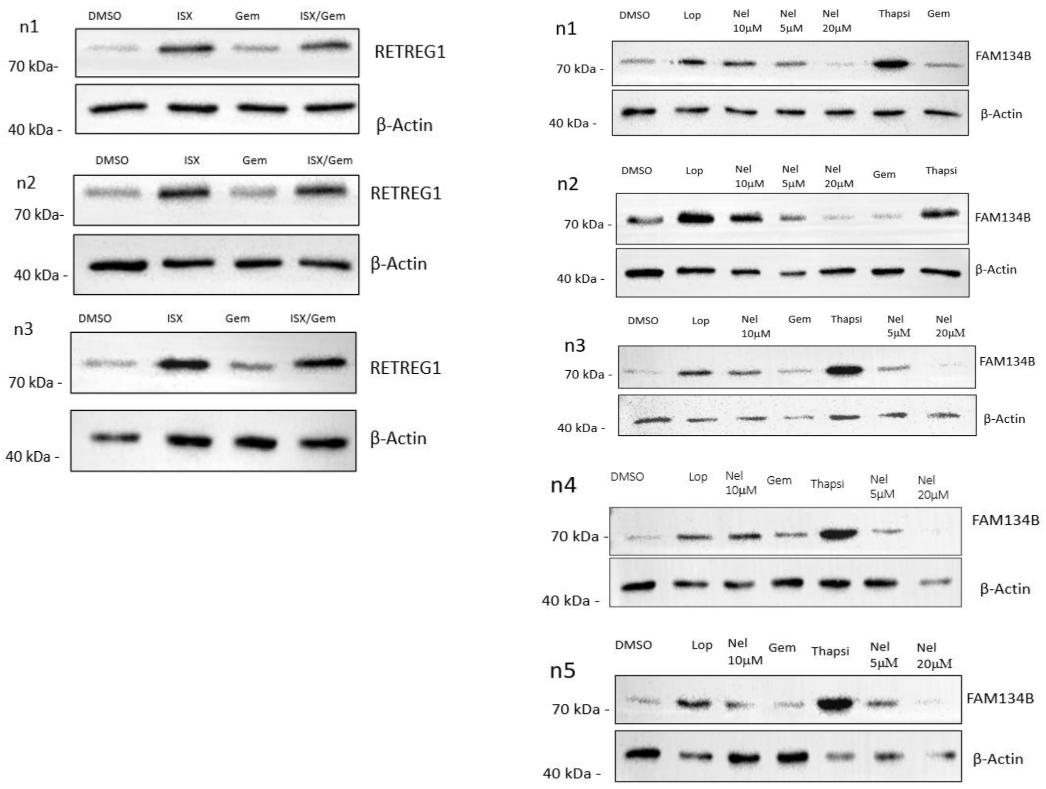

Supplement: Supplementary file 1 — Supplementary Material 1 [file 12964_2025_2198_MOESM1_ESM.pdf]
